# Evolutionary-inspired probabilistic search for enhancing sampling of local minima in the protein energy surface

**DOI:** 10.1186/1477-5956-10-S1-S5

**Published:** 2012-06-21

**Authors:** Brian S Olson, Amarda Shehu

**Affiliations:** 1 Department of Computer Science, George Mason University, 4400 University Dr., Fairfax, VA, 22030, USA; 2 Department of Bioinformatics and Computational Biology, George Mason University, 4400 University Dr., Fairfax, VA, 22030, USA; 3 Department of Bioengineering, George Mason University, 4400 University Dr., Fairfax, VA, 22030, USA

## Abstract

**Background:**

Despite computational challenges, elucidating conformations that a protein system assumes under physiologic conditions for the purpose of biological activity is a central problem in computational structural biology. While these conformations are associated with low energies in the energy surface that underlies the protein conformational space, few existing conformational search algorithms focus on explicitly sampling low-energy local minima in the protein energy surface.

**Methods:**

This work proposes a novel probabilistic search framework, PLOW, that explicitly samples low-energy local minima in the protein energy surface. The framework combines algorithmic ingredients from evolutionary computation and computational structural biology to effectively explore the subspace of local minima. A greedy local search maps a conformation sampled in conformational space to a nearby local minimum. A perturbation move jumps out of a local minimum to obtain a new starting conformation for the greedy local search. The process repeats in an iterative fashion, resulting in a trajectory-based exploration of the subspace of local minima.

**Results and conclusions:**

The analysis of PLOW's performance shows that, by navigating only the subspace of local minima, PLOW is able to sample conformations near a protein's native structure, either more effectively or as well as state-of-the-art methods that focus on reproducing the native structure for a protein system. Analysis of the actual subspace of local minima shows that PLOW samples this subspace more effectively that a naive sampling approach. Additional theoretical analysis reveals that the perturbation function employed by PLOW is key to its ability to sample a diverse set of low-energy conformations. This analysis also suggests directions for further research and novel applications for the proposed framework.

## Background

Characterizing the three-dimensional structures that protein molecules employ to carry out their biological activity in a living cell remains a central problem in computational structural biology [1]. Despite the challenges that this problem raises for computation, elucidating these structures is important. Proteins are ubiquitous biological molecules and play a critical role in many cellular processes. Moreover, there is a strong relationship between structure and biological function in protein molecules; proteins employ specific structures and often transition between them to interact with other molecules in cells [2].

Many experimental techniques, such as X-ray crystallography, nuclear magnetic resonance, and cryo-electron microscopy can elucidate one or a few structures populated under physiologic conditions. These techniques, however, cannot access the entire subspace of three-dimensional arrangements (also referred to as conformations) that are available to the chain of amino acids in a protein molecule under physiologic conditions. Accessing this subspace is important, as experiment, theory, and computation show that proteins are not rigid molecules but can employ internal motions to populate different conformations and modulate biological function [3-7]. Obtaining a representative view of the conformations available to a protein molecule under physiologic conditions presents an opportunity not only to improve our understanding of the structure-function relationship in proteins, but also to advance the development of synthetically engineered proteins, improve our models of protein ligand docking for drug development, and assist in the prediction of protein-protein interactions in supramolecular assemblies [8-10].

**Figure 1:**
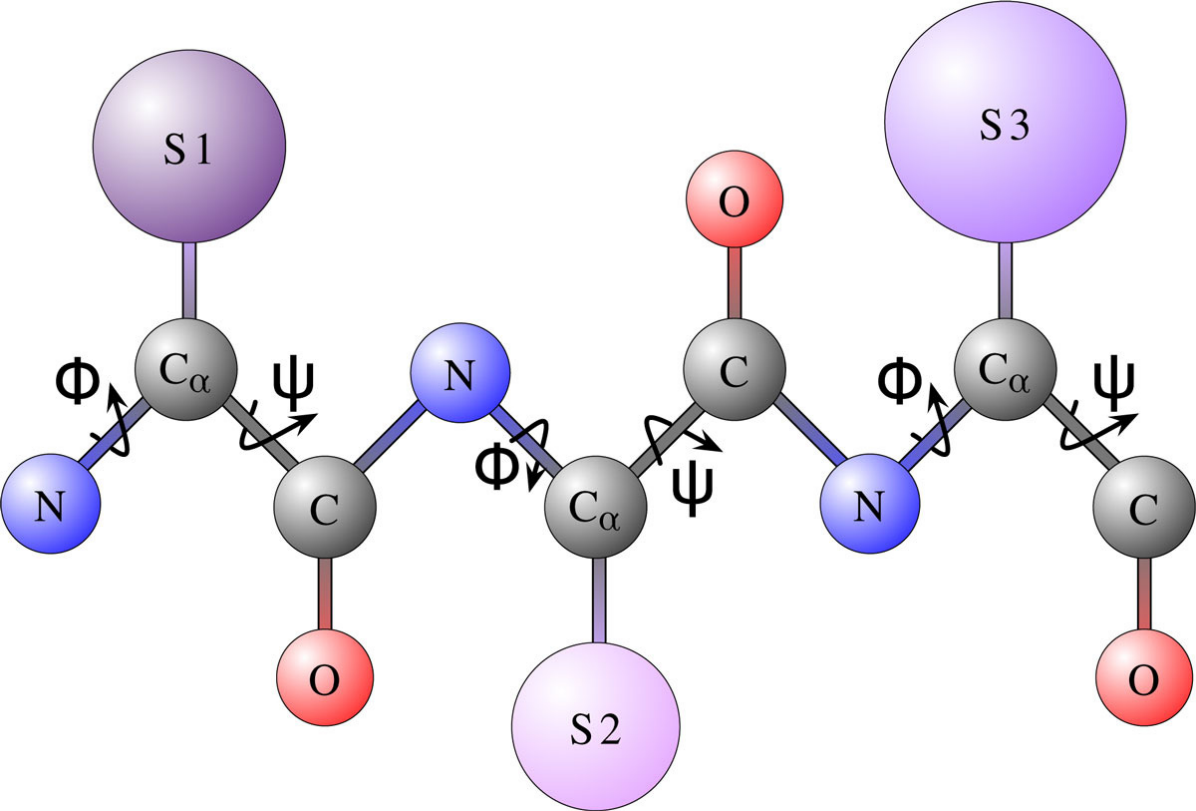
**A short protein chain of 3 amino acids is shown for the purpose of illustrating protein geometry**. All amino acids share a common set of atoms, *N *(in blue), *C_α _*(in gray), *C *(in gray), and *O *(in red), known as the backbone atoms. The set of atoms that makes an amino acid unique and confers to it a specific type is known as the side chain. Side chains for the shown chain are encapsulated in the violet spheres labeled *S*_1 _through *S*_3_. There are 20 different types of naturally-occurring amino acids. Side chains dangle off the backbone chain that connects the backbone atoms of consecutive amino acids. There are two backbone dihedral angles, *φ *and *ψ*, per amino acid. These angles are annotated over the shown backbone chain.

Computational methods present attractive complimentary approaches to experimental techniques for elucidating the conformations available to a protein chain under physiologic conditions. Elucidating conformations that are relevant for function is challenging. The space of conformations available to a protein chain is vast and high-dimensional. Even when foregoing some detail and modeling only the backbone atoms of a protein chain through the *ϕ *and *ψ *dihedral angles (these angles are illustrated in Figure 1 on a short protein chain), the number of angles needed to represent a conformation for a chain of *n *amino acids is 2*n*; the ensuing conformational space is 2*n*-dimensional.

As biological systems, protein molecules are characterized by physics-based energetic interactions. The sum of interatomic interactions in a conformation yields the inner free energy or the potential energy of a conformation. Associating this energy with each conformation of the protein conformational space reveals a multi-dimensional funnel-like energy surface underlying the protein conformational space [11-13]. The funnel essentially encapsulates the energetic bias that drives the protein chain to organize itself into lower-energy conformations [14]. This funnel-like energy surface, however, is not smooth but rich in local minima. Accessing conformations that are relevant for biological activity entails exploring the vast conformational space in search of the lowest-energy regions in the underlying energy surface. Conformational search methods often attempt to simplify the conformational space and energy surface that they navigate in search of low-energy conformations. Two popular strategies for this purpose are coarse-grained representations (and coarse-grained energy functions) and Fragment-based Assembly (FA), detailed in the Related work section below. While coarse-grained representations reduce the amount of detail needed to represent a conformation, and hence lower the dimensionality of the conformational space, FA essentially discretizes the underlying conformational space and allows assembling new conformations with structural pieces extracted from known protein structures.

Some of the most effective methods encapsulate the two strategies listed above in the context of probabilistic search. These methods often follow a two-stage exploration template [8,15-19]. Stage one explores the conformational space at a coarse-grained level of detail with the goal of obtaining a broad view of the conformational space. This is often implemented by launching many Metropolis Monte Carlo (MMC) or Molecular Dynamics (MD) trajectories to collect a large set of low-energy conformations. The exploration is then suspended, and a detached analysis identifies a subset of conformations from which further trajectories are launched in stage two of the exploration [8].

The search techniques typically employed in stage one do not explicitly sample local minima. Rather, the goal is to obtain a large number of low-energy conformations. It is the task of the analysis that follows stage one to group the obtained low-energy conformations by geometric similarity to reveal local minima. Because the analysis is detached from the exploration, many of the independent trajectories launched in stage one may converge to similar regions of the conformational space. A new search framework introduced by our lab makes the analysis part of the exploration itself in order to remedy this issue and adaptively guide the search towards under-sampled low-energy regions [20,21]. However, this framework also does not explicitly sample local minima during its search of the conformational space.

This paper introduces a probabilistic search framework to explicitly sample local minima in the protein energy surface. We refer to the framework as Protein Local Optima Walk (PLOW) from now on. Unlike other conformational search methods, PLOW does not waste computational resources to obtain a broad view of the conformational space and rely on further analysis to elucidate interesting low-energy regions. Instead, PLOW focuses its sampling of the conformational space on low-energy local minima, essentially obtaining a discrete representation of the protein conformational space relevant for function through a set of conformations that map to low-energy local minima in the underlying energy surface. By effectively using computational resources to essentially map the conformational space through the underlying set of low-energy local minima, PLOW allows accessing conformations relevant for function.

PLOW bears some resemblance to basin hopping techniques that modify an MMC or an MD trajectory to jump between local minima in the energy surface [22]. However, PLOW conducts a more effective exploration, as it follows a unifying search framework popular in the evolutionary computation community, Iterated Local Search (ILS) [23,24]. PLOW incorporates algorithmic ingredients of ILS and MMC and employs both FA and a coarse-grained representation (and coarse-grained energy function) in order to effectively sample conformations residing in low-energy local minima.

This paper first provides a focused review of relevant related work in the following section. Details on PLOW are related in the Methods section. The following Results section evaluates various components of PLOW's performance on 15 diverse protein systems. Specific experiments presented in this section compare PLOW to state-of-the-art conformational search methods. The analysis provides both an experimental basis for PLOW's success and allows identifying areas for further work. The conclusions section presents some of these directions for further research.

### Related work

A popular strategy that simplifies the conformational space and is commonly used by two-stage exploration-based methods employs coarse-grained representations of the protein chain. These representations reduce the amount of information needed to represent a conformation. The backbone representation described in the Background section above, which essentially maintains only the *ϕ *and *ψ *angles as parameters, is one such example. Coarse-grained energy functions accompany the coarse-grained representations to associate potential energies with computed conformations. All practical protein energy functions are semi-empirical and introduce potential distortions to the true energy surface by removing or introducing local minima. However, extensive research has gone into developing state-of-the-art coarse-grained energy functions and showing that they are effective for protein conformational search [25]. Another effective strategy for simplifying the conformational space is known as FA. FA essentially allows computing new protein conformations by obtaining values for 2*k *backbone dihedral angles at a time, where *k *is the number of amino acids in a fragment of the protein chain. Rather than sampling conformations one backbone dihedral angle at a time, FA discretizes the underlying conformational space by providing a limited set of different (angular) configurations for each fragment of *k *consecutive amino acids that can be defined over a given protein chain. The idea is to essentially assemble new conformations of a protein chain with structural pieces that are already available in known biologically-active structures deposited in protein structure databases. The pieces are extracted from functionally-relevant protein structures deposited by experimentalists in databases like the Protein Data Bank (PDB) [26]. These pieces are stored in a library as angular configurations indexed by the amino-acid sequence of the fragment in the protein chain from which they were extracted.

Conformational search algorithms that employ the two-stage exploration template described in the Backgrounds section often have as their driving goal the ability to recover a representative (native) structure assumed by a protein sequence under physiologic conditions for the purpose of biological activity. This driving goal often limits application of these methods to proteins where conformations populated by a protein molecule for the purpose of function are essentially fluctuations around a unique representative structure (the case for many small- to medium-size proteins). Given this driving application, these methods do not have to obtain a comprehensive view of the conformational space accessible for function. However, stage one of the exploration aims to obtain a broad view of this space in order to increase the probability that the sought-after native structure can be reached from at least one of the local minima populated in stage one with further search in stage two of the exploration.

Studies have shown that the probability to recover the native structure in stage two of the exploration increases if a few local minima are captured in the vicinity of the native structure [8]. There is no guarantee, however, that the independent search trajectories in stage one of the exploration will capture the relevant minima and not converge to a limited subset of nearby regions in the conformational space. An iterative approach is proposed in [16] to identify promising regions for further search early on. Essentially, the two stages of the exploration are interlaced. Stage one of the exploration is conducted at a coarse-grained level of detail, followed by analysis that identifies promising regions in this space. Short trajectories are conducted in greater, atomistic, detail in order to further explore select regions and distinguish those that represent local minima and are worth investigating further. This approach allows re-apportioning computational resources by essentially refocusing the exploration in stage one to interesting regions of the energy surface.

The idea of guiding search to promising regions is also incorporated in the FeLTr probabilistic search framework proposed by our laboratory [20,21]. FeLTr incorporates analysis in the search itself in order to bias the search away from redundant conformations in terms of energy and geometry. Instead of launching independent search trajectories, FeLTr grows a search tree in the conformational space. The tree grows by expanding selected conformations with short MMC trajectories and maintains a representative ensemble of previously visited conformations in memory. Selection from this ensemble is biased towards low-energy conformations in under explored regions of the conformational space. In this way, FeLTr dynamically redirects computational resources at the global level to ensure a degree of geometric diversity in its conformational sampling. Recent work shows that FeLTr is more effective at sampling low-energy conformations than independent MMC search trajectories [20,21,27,28].

Both FeLTr and the two-stage exploration-based methods summarized above do not explicitly sample local minima, but rather rely on clustering-based analysis to filter their results down to a subset of conformations which hopefully capture low-energy local minima that can drive further exploration to the sought-after native structure. In broader applications, it is important to obtain a broad view of the energy surface relevant for biological activity and map the low-energy local minima in this surface. For instance, many studies in computational biology and chemistry focus on sampling low-energy local minima by implementing basin/minima hopping techniques [22,29-31]. Essentially, the temperature schedule is adjusted in an MMC or MD search trajectory in order to allow these techniques to populate a local minimum (accomplished by lowering the temperature of the simulation) and then escape it (accomplished by raising the temperature). This process is repeated to allow the search trajectory to move from one local minimum to the next. Basin hopping has been effectively applied to map the protein energy surface for small proteins [29-31]. However, these approaches employ all-atom detail, and their computational complexity has limited their application to small proteins.

The existing minima hopping techniques in the computational biology and chemistry communities can be seen as specific realizations of the ILS evolutionary search framework. ILS is a trajectory-based version of a class of evolutionary search algorithms referred to as "memetic" algorithms. Memetic algorithms employ a local search algorithmic component to optimize points that are sampled by a global search algorithmic component. The interlacing of local and global search allows "memetic" algorithms to effectively sample local minima in a complex non-linear solution space, such as the rugged funnel-like energy surface associated with protein molecules. Not surprisingly, there has been extensive work on applying memetic evolutionary approaches to the problem of finding the protein native structure [32-35]. However, these studies use overly simplified representations, focus solely on optimization of an objective function, and fail to compare obtained conformations with with experimentally-available native structures.

The PLOW framework presented in this paper combines cutting-edge stochastic optimization strategies from the evolutionary computation community with established strategies in computational structural biology that simplify the protein conformational space and associated energy surface. PLOW offers a successful realization of ILS for obtaining a map of the lowest-energy local minima in the protein energy surface, which are relevant for biological activity. In evolutionary computing terminology, PLOW combines global search with an exploitative local search. The global search allows PLOW to explore the breadth of the energy surface, biasing towards lower-energy regions, while the local search optimizes each exploration at the global level to the closest low-energy local minimum. By interlacing global and local search, PLOW is able to more effectively sample a wide range of lowest-energy local minima. Details on PLOW are now related in the Methods section.

## Methods

The goal of the PLOW framework proposed in this paper is to obtain a discrete representation of the protein conformational space through a set of conformations that map to low-energy local minima in the underlying protein energy surface. PLOW is a novel evolutionary-inspired probabilistic search framework that incorporates algorithmic ingredients from ILS and MMC to explore the space of local minima by effectively sampling conformations residing in these minima. Before relating details on PLOW, we provide context and an overview of the main algorithmic ingredients of the framework.

### Focus on low-energy local minima

Unlike other conformational search methods that aim to obtain a broad view of the conformational space and rely on later analysis to identify any low-energy local minima that may have been captured, PLOW focuses its sampling explicitly on local minima. This focus is warranted for the following two reasons.

First, the protein conformational space is vast and high-dimensional. Even with techniques, such as FA and reduced representations (reviewed in Related work), that aim to simplify and lower the dimensionality of the effective conformational space, the search space remains vast. PLOW addresses this challenge by focusing its sampling on conformations residing in low-energy local minima, essentially obtaining a discrete representation of the relevant conformational space. Second, the conformational space is dominated by high-energy conformations. Naive probabilistic search techniques spend a significant portion of their time sampling these irrelevant conformations. PLOW essentially biases its exploration away from high-energy conformations by focusing its sampling of the conformational space on low-energy local minima. An important feature of PLOW is that the framework progressively strengthens its bias to guide its exploration towards lower-energy local minima.

### Sampling a local minimum

**Figure 2:**
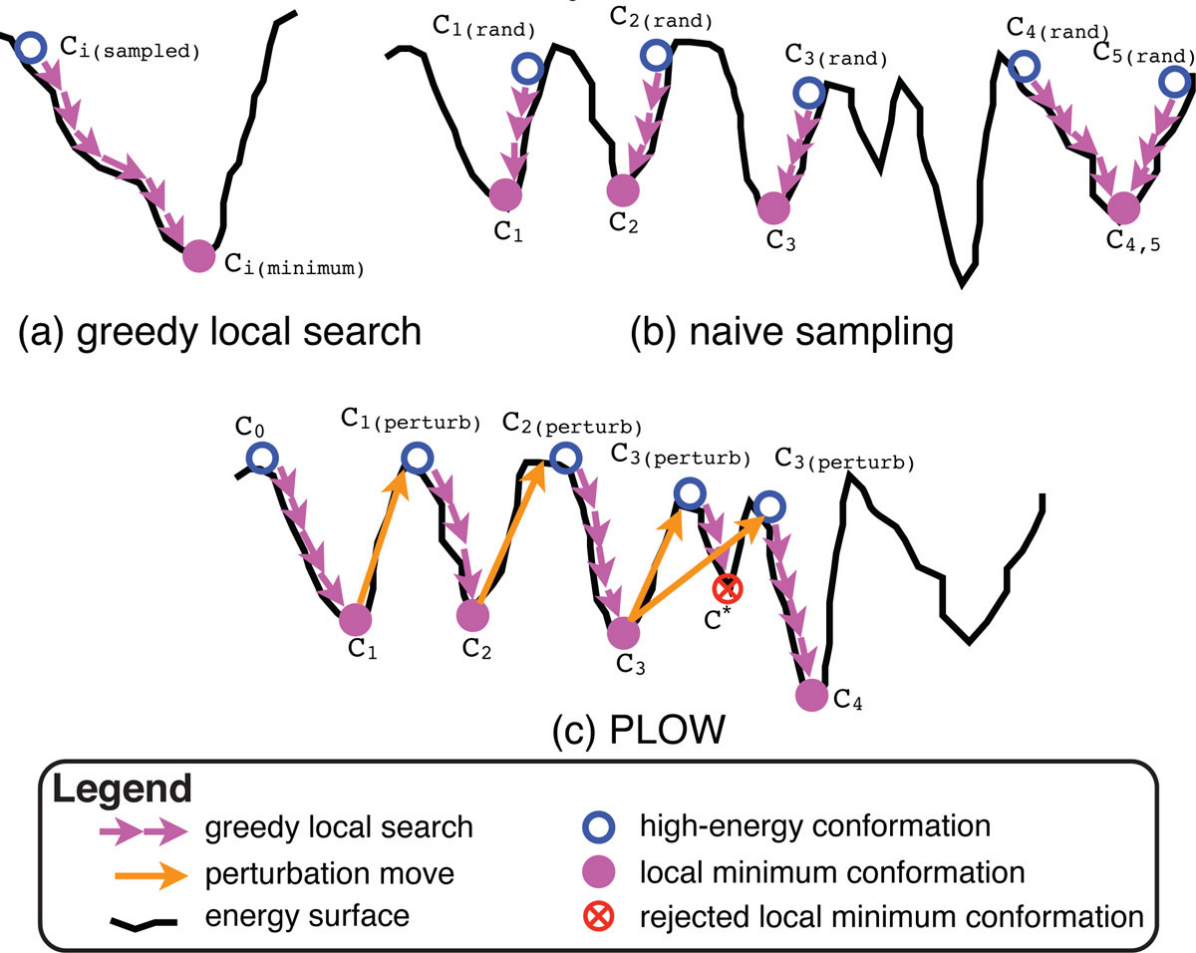
**The figure illustrates (a) greedy local search, (b) naive sampling, and (c) PLOW on a simplified energy surface**. (a) A sampled conformation *C_i_*_(sampled) _(empty blue circle) is mapped to the nearest local minimum *C_i_*_(minimum) _(solid purple circle) by a greedy local search (series of short purple arrows). (b) 5 points sampled at random (empty blue circles) by the naive sampling approach are each mapped to a nearby local minimum (solid purple circles) by a greedy local search (series of short purple arrows). (c) PLOW begins at *C*_0 _(leftmost empty blue circle). Through a series of perturbations (long orange arrows) and greedy local searches (short purple arrows), PLOW samples conformations representative of local minima (*C*_1 _through *C*_4_) in the energy surface.

An important question to address is how to explicitly sample a local minimum in the energy surface. PLOW addresses this by first sampling a conformation in the conformational space and then efficiently mapping that conformation to a nearby local minimum through a series of small modifications. PLOW only accepts modifications which lower the energy of the protein system under consideration in order to drive the trajectory of consecutively obtained conformations down towards a nearby local minimum. Figure 2(a) illustrates this process by showing how a series of accepted modifications maps a sampled conformation to a nearby local minimum.

By insisting that each modification lower the energy, PLOW essentially implements greedy search. Greedy search is more preferable to other alternatives, including conjugate gradient descent, MD, or MMC, because it is more efficient at finding the nearest local minimum. Conjugate gradient descent and MD can be rather slow by following the negative gradient of the energy function. Employing MMC would require controlling its effective temperature in order to tune the MMC behavior from a technique in search of the global minimum to a technique in search of the nearest local minimum. The greedy search employed by PLOW to map a sampled conformation to a nearby local minimum is detailed in the Greedy search: mapping a conformation to a nearby local minimum section.

### A naive approach to sampling local minima

Equipped with a technique to map a conformation to its nearest local minimum, a trivial and naive approach can now be put together to sample local minima in the energy surface by essentially repeating the following two steps: (i) sample a conformation *C_i_*_(rand)_uniformly at random in the conformational space; (ii) map *C_i_*_(rand) _to a conformation *C_i _*that resides in the nearest local minimum. Figure 2(b) illustrates this process with 5 randomly sampled conformations (*C*_1(rand)_-*C*_5(rand)_) which are mapped to corresponding local minima (*C*_1_-*C*_5_). In the case of *C*_4(rand) _and *C*_5(rand)_, both randomly sampled conformations map to the same local minimum. The result of this naive approach is an ensemble of *C_i _*conformations representing sampled local minima in the protein energy surface. This naive approach is akin to a classic random search over the subspace of local minima. In fact, realizations of this approach can be found in computational structural biology, whether the focus is to model equilibrium fluctuations of loop fragments in protein structures [36,37] or to obtain equilibrium conformational ensembles of peptides and proteins [19,38]. It is important to note that different specific implementations can be found in literature for steps (i) and (ii). The Results section compares PLOW to an implementation of this naive approach that uses the same greedy search to map a conformation to its nearest local minimum, and the same energy function and reduced representation as PLOW for a direct comparison. In addition, since PLOW uses FA (details related below), *C*_rand _conformations in the naive approach are assembled through FA with random fragment configurations sampled from the fragment configuration library. Our results show that this simple naive approach can be effective on small proteins, as also supported by computational structural biology work that implements this approach on short loop fragments and small peptides and proteins [19,36-38]. However, on larger systems the ability to jump from one local minimum to the next allows PLOW to more efficiently sample low-energy local minima.

The rest of the Methods section now relates details on the proposed PLOW framework.

### Effective sampling of local minima: Protein Local Optima Walk (PLOW)

The naive approach above samples conformations in the subspace containing only local minima. This subspace, however, while dramatically reduced in size compared to the entire protein conformational space, is still too large to capture through essentially random search. Moreover, not all local minima are interesting. An effective search should progressively guide its exploration to lower-energy local minima if its focus is on obtaining physically-relevant conformations. PLOW achieves this through a trajectory-based exploration of the subspace of local minima that is progressively steered towards lower-energy local minima. The first conformation in the PLOW trajectory is obtained after applying greedy search to a fully extended initial conformation. What distinguishes PLOW from the naive sampling approach described above is that the current sampled local minimum (the result of step (ii) above) determines, to a great extent, the conformation employed (instead of step (i) above) to obtain the next local minimum in the trajectory. This dependence is implemented through a special perturbation move which essentially allows PLOW to jump out of a current local minimum and so obtain a new initial conformation from where to start its search of another nearby local minimum with greedy search. The perturbation move in PLOW is inspired by ILS, an evolutionary search framework [23,24]. In summary, the conformations in the trajectory are obtained as a series of perturbation moves followed by greedy search. It is important to note that PLOW progressively guides the trajectory towards lower-energy minima through the Metropolis criterion traditionally employed in an MMC search.

Figure 2(c) illustrates the essential process in PLOW on a simple two-dimensional energy surface. In the illustration, PLOW begins at a fixed point, *C*_0 _(shown as the empty blue circle on the far left), which is mapped to a local minimum, *C*_1_, by the greedy search (shown as a series of short purple arrows). PLOW then escapes its current local minimum, *C*_1_, through a perturbation move (shown as a long orange arrow). The resulting conformation, *C*_1(perturb)_, is again mapped onto a nearby local minimum, *C*_2_, through the greedy search. Now a decision must be made to accept *C*_2 _as the new state of the search trajectory. In Figure 2(c), both *C*_2 _and *C*_3 _are accepted; C* is rejected, however, because it has a much higher energy and fails the Metropolis criterion. In this case, PLOW remains at *C*_3 _and performs a second perturbation followed by greedy search to reach *C*_4_.

The remaining sections of Methods now describe in detail the greedy search, the perturbation move, and the acceptance criterion employed to guide the trajectory towards lower-energy local minima.

### Greedy search: mapping a conformation to a nearby local minimum

A greedy search maps a conformation onto a nearby local minimum in the energy surface through a series of small modifications. A modification consists of replacing the configuration (6 backbone dihedral angles) of a fragment of three consecutive amino acids (trimer) in the current conformation with a configuration sampled from a configuration library. This is known as FA, and a description can be found below. A modification that does not result in a lower-energy conformation is discarded, and another modification is performed. Successful modifications result in consecutive conformations that lower potential energy. The greedy search stops when *k *consecutive modifications fail to result in a lower energy, indicating the presence of a local minimum. The value of *k *is set to the length of the target protein (number of amino acids). The greedy search encapsulates the working definition of a local minimum. In essence, the value of *k *defines how deeply a local minimum is probed. Exhaustively testing for the presence of a local minimum is prohibitive, as it requires thousands of energy evaluations. Our approximation of a local minimum here does not waste resources by unnecessarily probing down to the true local minimum. Moreover, this approach is sufficient when empirical, potentially coarse-grained, energy functions are employed to probe an effective, rather than the true, energy surface. Our Results section shows, for instance, that the native structure of a protein is often found somewhere above the basin of the energy surface that can be probed with a coarse-grained energy function.

### Perturbation move: jumping out of a local minimum

The goal of a perturbation is to allow PLOW to jump out of the current local minimum so another nearby local minimum can be sampled. The perturbation needs to make a move that is not too small, so PLOW can jump out of a local minimum, but is also not too large, so PLOW can still benefit from knowledge of the previous local minimum and not devolve into random search.

The perturbation move is implemented by replacing the configuration of a selected trimer over the protein chain in the conformation representing the currently sampled local minimum with a configuration sampled from the trimer configuration library. This implementation is sufficient to obtain a high-energy conformation that takes PLOW out of the current local minimum. The reason is that low-energy conformations tend to be compact and leave little room for movement in their backbone chain without raising potential energy. The conformation obtained after the perturbation move will share nearly all of its local structural features with its parent conformation (the one residing in the local minimum), but the new conformation will have a much higher energy and a significantly altered overall global structure.

Given that the perturbation move results in a high energy, the greedy search described above can then optimize the perturbed conformation *C_i_*_(perturb) _and map it to one of many distinct local minima *C_i_*_+1_, leaving little chance that the mapping will return PLOW to its previously sampled local minimum *C_i _*(see Figure 2(c) for an illustration). However, because most of the local structural features of *C_i _*are maintained in the perturbed conformation *C_i_*_(perturb) _, the greedy search will benefit from such knowledge and be able to map *C_i_*_(perturb) _to a nearby local minimum *C_i_*_+1_. For these reasons, a single trimer configuration replacement serves as an effective perturbation move.

### Acceptance criterion

After each *C_i_*_(perturb) _has been mapped to a nearby local minimum *C_i_*_+1 _by the greedy search, PLOW decides whether or not to accept *C_i_*_+1 _and add it to its trajectory or remain at *C_i_*. PLOW employs the Metropolis criterion to make this decision [39]. According the Metropolis criterion, *C_i_*_+1 _will be accepted if it has lower energy than *C_i_*. Otherwise, it will be accepted with probability *e*^-ΔE*β^, where Δ*E *is the energetic difference from *C_i_*_+1 _to *C_i_*, and *β *is a scaling parameter that depends on an employed effective temperature. In this implementation, this parameter is set so that 10 kcal/mol energetic increases are accepted with probability 0.1. (We have employed the same parameter value in previous work [21]).

### Representation, fragment-based assembly, and energy function

PLOW employs a coarse-grained representation, modeling only two backbone degrees of freedom per amino acid. An individual conformation is represented as a vector of 2*n *dihedral bond angles, where *n *is the number of amino acids. Modifying a conformation employs FA [40]. The idea is to associate physically-relevant configurations with fragments of consecutive amino acids in a protein chain. All native protein structures in structure databases such as the PDB are analyzed, and fragment configurations are excised from these structures and stored in a fragment configuration library. A single fragment configuration replacement consists of first selecting, at random, a position in a given protein chain and then selecting, at random, a configuration stored for that fragment from the configuration library. Essentially, the bond angles from the selected configuration are copied into the vector representation of the current conformation, resulting in a new conformation. Here we use fragments of length three, trimers, and a trimer configuration library constructed as in our previous work [20].

The energy function employed to evaluate each conformation is a modified implementation of the Associative Memory hamiltonian with Water (AMW) [41]. The energy is the sum of the non-local terms *E_Lennard−Jones_, E_H−Bond_, E_contact_, E_water_, E_burial_*, and *E_Rg_*. Local terms are not modeled because local interactions are already near ideal levels in conformations assembled with fragment configurations extracted from the PDB. The *E_contact_, E_water_*, and *E_burial _*terms simulate interactions due to solvation in water. The *E_Rg _*term penalizes non-compact conformations. Additional details on the energy function can be found in our previous work [15] and various applications of it in the context of conformational search algorithms by various labs [20,21,42-45]

## Results and discussion

### Experiments conducted to study performance

We conduct the following set of experiments to analyze the performance of the PLOW framework: (I) Analysis of local minima: this first experiment explores the accuracy of the employed AMW energy function with respect to local minima in order to determine the extent to which this energy function allows probing a selected true minimum in the protein energy surface. (II) Comparison of PLOW to the naive approach: this second experiment compares PLOW to the naive approach in sampling local minima, as described in the Methods section. The goal is to directly measure the effect of the trajectory-based exploration in PLOW on enhancing the sampling of lower-energy local minima. (III) Comparison of PLOW to FeLTr: this third experiment compares PLOW to the tree-based exploration in FeLTr in terms of the proximity of obtained conformations to a known native structure of a protein system under investigation. An additional modification is conducted on the FeLTr framework in order to provide a more direct comparison with PLOW, resulting in FeLTr*. (IV) To place PLOW in a broader context with respect to other conformational search algorithms, this experiment compares PLOW to state-of-the-art search algorithms of other groups. (V) Finally, an interesting analysis is conducted in the final experiment that correlates the size of the perturbation move in PLOW to its ability to obtain conformations in close proximity to the known native structure.

### Systems of study

All the experiments listed above are conducted on a broad set of 15 protein systems. These systems, listed in Table 1, range from 61 to 123 amino acids in length and represent a diverse set of *α, β*, and *αβ *topologies. Many of these systems have been studied by other conformational search algorithms and so allow a direct comparison of PLOW with results published by other groups.

**Table 1:** Target proteins

	PDB id	Length	Fold	% *α*	% *β*	lRMSD of nearest local minimum to native (Å)
1	1DTDB	61	*αβ*	14	45	1.3
2	1ISUA	62	*αβ*	14	19	0.4
3	1C8CA	64	*αβ*	21	48	1.5
4	1SAP	66	*αβ*	30	43	2.9
5	1HZ6A	67	*αβ*	29	38	2.0
6	1WAPA	68	*β*	0	56	1.5
7	1FWP	69	*αβ*	15	12	0.4
8	1AIL	70	*α*	80	0	2.5
9	1AOY	78	*αβ*	41	10	3.9
10	1CC5	83	*α*	46	2	1.5
11	2EZK	93	*α*	63	0	2.9
12	1HHP	99	*αβ*	7	48	2.2
13	2HG6	106	*αβ*	28	17	2.5
14	3GWL	106	*α*	69	0	2.7
15	2H5ND	123	*α*	65	1	1.7

The PDB id, length, and fold are given for each of the 15 target proteins. Columns 5 and 6 represent the percentage of amino acids which are *α *helices and *β *sheets, respectively, for each target. Column 7 gives the lRMSD between the native structure and the closest local minimum found when performing multiple greedy local searches starting from the native structure.

### Experimental setup

PLOW samples local minima under a fixed budget of 10, 000, 000 energy function evaluations. This decision is made for the following reason. Conformational search algorithms spend significant time computing potential energies (for instance, PLOW and FeLTr spend over 90% of their CPU time). The computational cost of an energy function is related to the length of a protein and so increases with protein length. Therefore, holding the number of energy evaluations constant (rather than total CPU time) ensures a fair comparison between all methods across a broad range of protein lengths. Moreover, since PLOW and FeLTr use the same energy function, fixing the number of energy evaluations masks any differences in implementation efficiency. In terms of time, this number of energy evaluations takes about 2-4 days of CPU user time on a 2.66GHz Opteron processor with 8GB of memory, depending on the length of the protein system under investigation.

### Performance metrics

While PLOW can be employed in different application settings, the one on which we choose to study and measure the performance of PLOW in this paper is the ability to reproduce the known native structure of a protein system. Essentially, obtained conformations are compared to the native structure available for a protein system in the PDB [26]. The comparison employs the least Root Mean Square Deviation (lRMSD) metric which measures the a weighted Euclidean distance between corresponding N, *C_α_*, C, and O atoms in two aligned conformations. The lowest lRMSD over PLOW-obtained conformations is reported and compared to that obtained or reported by other conformational search algorithms.

#### I. Analysis of local minima

Empirical energy functions, such as the ones available to evaluate energy on protein chains longer than 2-3 amino acids, are known to contain errors due to their approximation of potential energy. They can be particularly insensitive in lower-energy regions of the protein energy surface and may not allow probing certain true minima in this surface [25]. Therefore, it is important to evaluate the extent to which the AMW energy function employed by PLOW allows probing local minima. We evaluate this in the context of our chosen application of PLOW in this paper, the reproduction of the native structure. This structure should reside in a local minimum even when employing a coarse-grained energy function like AMW. Specifically, we evaluate whether the native structure of each of the 15 protein systems studied in this paper resides in a local minimum of the energy surface probed by AMW. Since PLOW samples local minima, this analysis allows determining the extent to which PLOW is able to reproduce the native structure.

The following experiment is conducted. Repeated greedy local searches are initiated from the known native structure of a protein system. Greedy search is implemented as described in Methods, except each search runs for a fixed 100,000 iterations. The distance, in terms of lRMSD, between the native structure and the nearest local minimum discovered by each greedy search is then recorded. The lowest distance found by the repeated searches is reported in column 7 in Table 1. For 14 out of the 15 protein systems investigated in this study, this distance is less than 3Å in lRMSD. This is a small distance that can be typically overcome by an all-atom energetic refinement [46]. This distance suggests that for 14 out of the 15 protein systems, the native structure resides near a local minimum, and so can be found even when sampling only local minima, as PLOW does. It is interesting to note that the only protein system in which the native structure is more than 3Å away from the nearest local minimum is also one out of two cases where FeLTr outperforms PLOW in terms of lowest lRMSD to the known native structure. The described analysis provides an independent means by which to measure the extent to which the energy function allows PLOW to succeed or fail in reproducing the native structure of a protein system.

#### II. Comparison of PLOW to a naive sampling of local minima

The Methods section describes a naive sampling of local minima, where essentially conformations are sampled at random in the conformational space and then mapped to nearby local minima with the greedy search of PLOW. Comparison of PLOW to this naive approach allows evaluating the extent to which the ability to jump from one local minimum to the next through the use of the perturbation move improves the sampling of low-energy local minima. The conformation residing in a local minimum that has the lowest lRMSD is reported in Table 2 for both, PLOW and the naive sampling approach.

**Table 2:** Distance from the native structure

				local		avg (min) lowest lRMSD to native in Å				
	PDB id	len	fold	search len	PLOW	Naive Sampling	FeLTr	FeLTr*	Sosnick	Baker
1	1DTDB	61	*αβ*	160	7.0(6.3)	6.9(6.0)	7.7(6.8)	7.5(7.0)	6.5	5.7
2	1ISUA	62	*αβ*	173	6.1(6.0)	6.7(6.6)	6.6(6.3)	6.5(5.7)	6.5	6.9
3	1C8CA	64	*αβ*	199	7.3(6.9)	7.2(6.8)	6.8(6.0)	7.2(5.8)	3.7	5.0
4	1SAP	66	*αβ*	211	6.7(6.2)	7.0(6.3)	7.1(6.5)	7.3(6.8)	4.6	6.6
5	1HZ6A	67	*αβ*	182	6.3(6.1)	5.8(5.6)	6.7(6.6)	6.6(6.1)	3.8	3.4
6	1WAPA	68	*β*	199	7.4(6.9)	7.8(7.6)	8.1(7.3)	7.3(6.5)	8.0	7.7
7	1FWP	69	*αβ*	210	6.3(5.2)	6.5(6.0)	7.3(6.4)	7.1(6.8)	8.1	7.3
8	1AIL	70	*α*	237	2.8(2.0)	4.1(3.8)	4.8(4.5)	4.0(3.4)	5.4	6.0
9	1AOY	78	*αβ*	258	5.6(5.3)	6.3(5.8)	5.2(4.6)	5.8(5.2)	5.7	5.7
10	1CC5	83	*α*	274	5.7(5.4)	5.5(4.7)	6.2(5.6)	5.8(4.9)	6.5	6.2
11	2EZK	93	*α*	293	4.7(4.3)	4.9(4.5)	6.5(6.0)	6.0(4.7)	5.5	6.6
12	1HHP	99	*αβ*	306	10.2(9.7)	10.8(10.3)	11.2(10.0)	11.0(9.7)	NA	NA
13	2HG6	106	*αβ*	376	8.9(8.1)	9.7(9.2)	10.0(9.6)	9.7(9.0)	NA	NA
14	3GWL	106	*α*	375	4.3(3.7)	5.7(5.5)	6.6(5.7)	6.3(4.4)	NA	NA
15	2H5ND	123	*α*	482	7.3(6.8)	8.3(8.0)	9.0(8.5)	8.6(7.8)	NA	NA

The lowest lRMSD to the native structure achieved is shown for our new PLOW framework, the naive sampling approach, and our previously developed FeLTr framework as well as published results from the Sosnick [18] and Baker [47] research groups. The lRMSDs shown are the average over five runs, with the minimum of the five runs shown in parentheses. Column 5 shows the average number of iterations each PLOW greedy search ran for. FeLTr* represents the FeLTr framework using the value from column 5 as its MMC search length.

**Figure 3:**
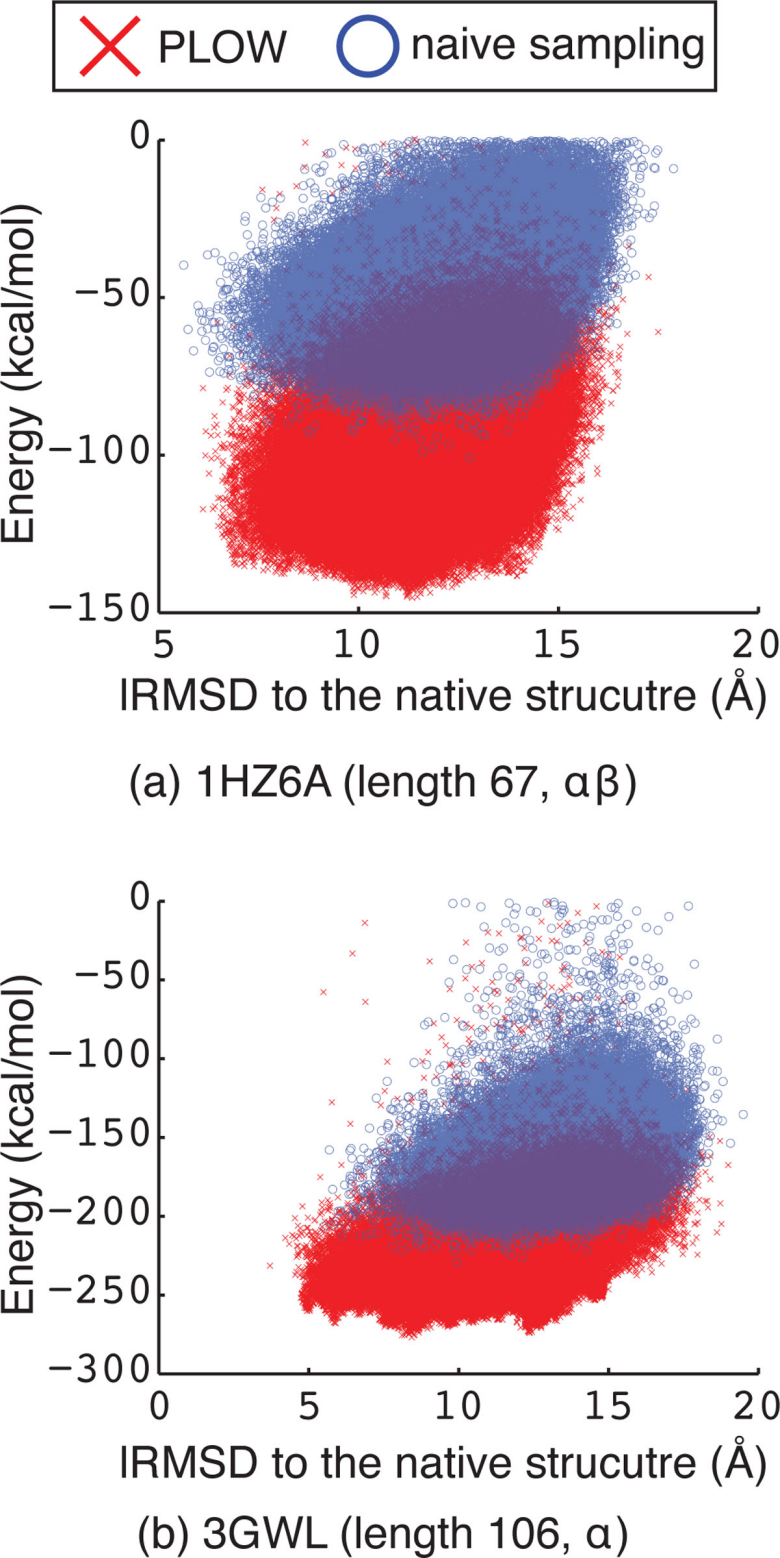
**Potential energy is plotted against lRMSD to the native structure for all conformations representative of sampled local minima**. Results obtained by the naive sampling approach (blue "o") are superimposed over those obtained by PLOW (red "x"). (a) shows results obtained for the protein system with PDB id 1HZ6A. (b) shows the results the protein system with PDB id 3GWL.

In order to conduct a fair comparison and account for the stochasticity in these conformational search approaches, both PLOW and the naive sampling approach are run 5 times on each of the 15 protein systems studied here. Table 2 reports the minimum and average lowest lRMSD over each of the 5 runs under each approach (PLOW in column 6 and the naive sampling approach in column 7). The results show that the naive sampling approach achieves similar minimum and average lowest lRMSDs to PLOW in many of the protein systems. However, on the longer protein chains, the performance of the naive sampling approach deteriorates. PLOW outperforms naive sampling by 0.5Å or more in 7 cases including the 4 longest proteins (see rows with PDB ids 1HHP, 2HG6, 3GWL, and 2H5ND in Table 2). This suggests that on the shorter chains naively sampling a high number of local minima can stumble across one near the native structure with a high probability. On the longer chains, the space of local minima grows, and a trajectory-based exploration like the one in PLOW is more effective at approaching the native structure. Looking at the lowest LRMSDs does not provide a full picture of the distribution of sampled conformations. Figure 3 plots the energy versus lRMSD to the known native structure for each conformation representative of a local minimum on two representative protein systems. The results obtained by the naive approach are superimposed over those obtained by PLOW.

Figure 3 shows that PLOW is able to reach significantly lower-energy minima than the naive sampling approach on both protein systems. A similar result is obtained on all of the systems studied in this paper (data not shown). Figure 3(a) shows that, for the smaller protein with PDB id 1HZ6A, the naive sampling approach stumbles upon a few conformations in closer proximity to the native structure than PLOW. If outliers are removed, however, both methods perform similarly well with respect to low lRMSD to the known native structure. Figure 3(b) shows that, for the larger protein with PDB id 3GWL, PLOW is able to sample conformations which are both lower in energy and closer to the native structure. This result holds even if outliers are removed.

#### III. Comparison of PLOW to FeLTr and FeLTr*

We now compare PLOW to FeLTr, a tree-based search framework developed in our lab [20]. Since FeLTr is a state-of-the-art probabilistic search framework that does not explicitly sample local minima, this comparison allows us to investigate the extent to which the explicit sampling of local minima in PLOW is more effective than the exploration in FeLTr. As the comparison of PLOW to the naive sampling approach above, the minimum and average lowest lRMSDs achieved over five runs of each framework are reported and compared in Table 2 (PLOW in column 6 and FeLTr in column 8).

Table 2 shows that PLOW outperforms FeLTr in every case, except the systems with native structure PDB ids 1AOY and 1C8CA. In 10 of systems, the difference is greater than 0.5Å lRMSD. PLOW significantly outperforms FeLTr by at least 1.0Å in key cases, including the longer proteins (PDB ids 2EZK, 1HHP, 2HG6, 3GWL, and 2H5ND); in the case of the protein with native structure PDB id 1AIL, PLOW finds a local minimum that is within 2Å lRMSD from the native. It is worth emphasizing that this is an impressive result. This protein is not small but 70 amino acids in length. Moreover, lRMSDs of 1 − 2Å are often obtained only by protocols after some form of all-atom energetic refinements on selected conformations, whereas search algorithms that employ coarse-grained energy functions often saturate at 4 − 5Å from the native structure. This result by PLOW suggests that the focus on local minima in PLOW allows effectively locating conformations very near the native structure.

We note that, in PLOW, the length of the greedy search is not determined a priori and can vary. In FeLTr, instead, the inner MMC trajectory that obtains a new conformations from a selected conformation in the FeLTr tree has a fixed length. In order to rule out the possibility that PLOW is merely benefiting from longer greedy searches, we modify FeLTr and obtain FeLTr* by extending the length of the MMC trajectory to the average greedy search length in PLOW. These average lengths are shown in Table 2, column 5. The results for FeLTr* in column 9 show that FeLTr* performs slightly better than FeLTr and is even comparable to PLOW in a few cases (proteins with native structure PDB ids 1ISUA, 1C8CA, 1HZ6A, 1WAPA, 1AOY, and 1CC5). On average, however, PLOW still outperforms FeLTr*, especially in the case of the five longer proteins with native structure ids 2EZK, 1HHP, 2HG6, 3GWL, and 2H5ND. This additional comparison confirms that there is a distinct advantage that the greedy search confers to PLOW. While the average length of the local search is the same between PLOW and FeLTr*, PLOW is able to vary this length as necessary to reach a local minimum.

#### IV. Comparison of PLOW to other state-of-the-art methods

Table 2 additionally compares PLOW to published results from other groups on 11 of the 15 protein systems studied here. Two state-of-the-art methods from the Sosnick [18] and Baker [47] groups are selected for this purpose. Results from these groups are shown in Table 2 in columns 10 and 11, respectively. PLOW outperforms these other methods by more than 0.5Å in 6 cases (proteins with native structure PDB ids 1ISUA, 1WAPA, 1FWP, 1AIL, 1CC5, and 2EZK), which include all three fold topologies and the longest of the 11 systems, as well. Of the remaining five proteins, PLOW performs worse in four cases (proteins with native structure PDB ids 1DTDB, 1C8CA, 1SAP, and 1HZ6). These results are expected, as the methods employ different energy functions and sampling techniques. However, the results show that PLOW achieves comparable results to state-of-the-art methods dedicated to reproducing the native structure of a protein system.

#### V. Perturbation analysis

This final experiment looks into the effect of the perturbation move in greater detail. We recall that a perturbation move helps PLOW to jump out of a local minimum represented by a conformation *C_i _*to obtain a conformation *C_i_*_(perturb)_. If the perturbation makes small moves in conformational space, the risk is that the subsequent application of greedy search to *C_i_*_(perturb) _will bring PLOW back to the same local minimum represented by *C_i_*. If, instead, the perturbation makes very large moves in conformational space, the subsequent application of greedy search to *C_i_*_(perturb) _will result in a minimum at *C_i_*_+1 _that is far away in conformational space from *C_i_*; the benefit of the trajectory-based exploration in PLOW will be lost, effectively deteriorating into a naive sampling of local minima.

In order to better understand the connection between the extent of the jump performed by the perturbation move and the success of PLOW in reproducing the native structure, we record the lRMSD between every *C_i _*and the *C_i_*_(perturb)_resulting after a perturbation move is applied to *C_i_*. Table 3 shows in column 6 the mean lRMSD distance over all perturbation moves in PLOW. Column 5 provides greater detail by showing that in only 25% or less of the perturbation moves, the lRMSD between *C_i _*and *C_i_*_(perturb)_, are less than 1Å. This suggests that the majority of perturbation moves are able to jump out a local minimum.

**Table 3:** Perturbation distance

				perturb distance		mean consecutive local
	PDB ID	Length	Fold	% < 1Å	mean (Å)	minima distance (Å)
1	1DTDB	61	*αβ*	5	6.0	7.2
2	1ISUA	62	*αβ*	10	5.6	6.5
3	1C8CA	64	*αβ*	18	5.5	6.3
4	1SAP	66	*αβ*	18	5.5	6.4
5	1HZ6A	67	*αβ*	11	5.0	6.0
6	1WAPA	68	*β*	6	6.4	7.9
7	1FWP	69	*αβ*	13	5.7	6.9
8	1AIL	70	*α*	25	4.5	4.5
9	1AOY	78	*αβ*	21	4.4	5.6
10	1CC5	83	*α*	23	4.8	6.2
11	2EZK	93	*α*	24	3.3	4.5
12	1HHP	99	*αβ*	6	7.2	9.5
13	2HG6	106	*αβ*	17	6.2	7.9
14	3GWL	106	*α*	24	4.2	6.1
15	2H5ND	123	*α*	24	4.6	6.9

The mean perturbation distance and the mean distance between consecutive local minima *C_i _*and *C_i_*_+1 _is given in columns 6 and 7, respectively. Column 5 represents the percent of *C_i_*_(perturb)_'s which are within 1Å lRMSD of *C_i _*and thus deemed to have not escaped the current local minimum.

**Figure 4:**
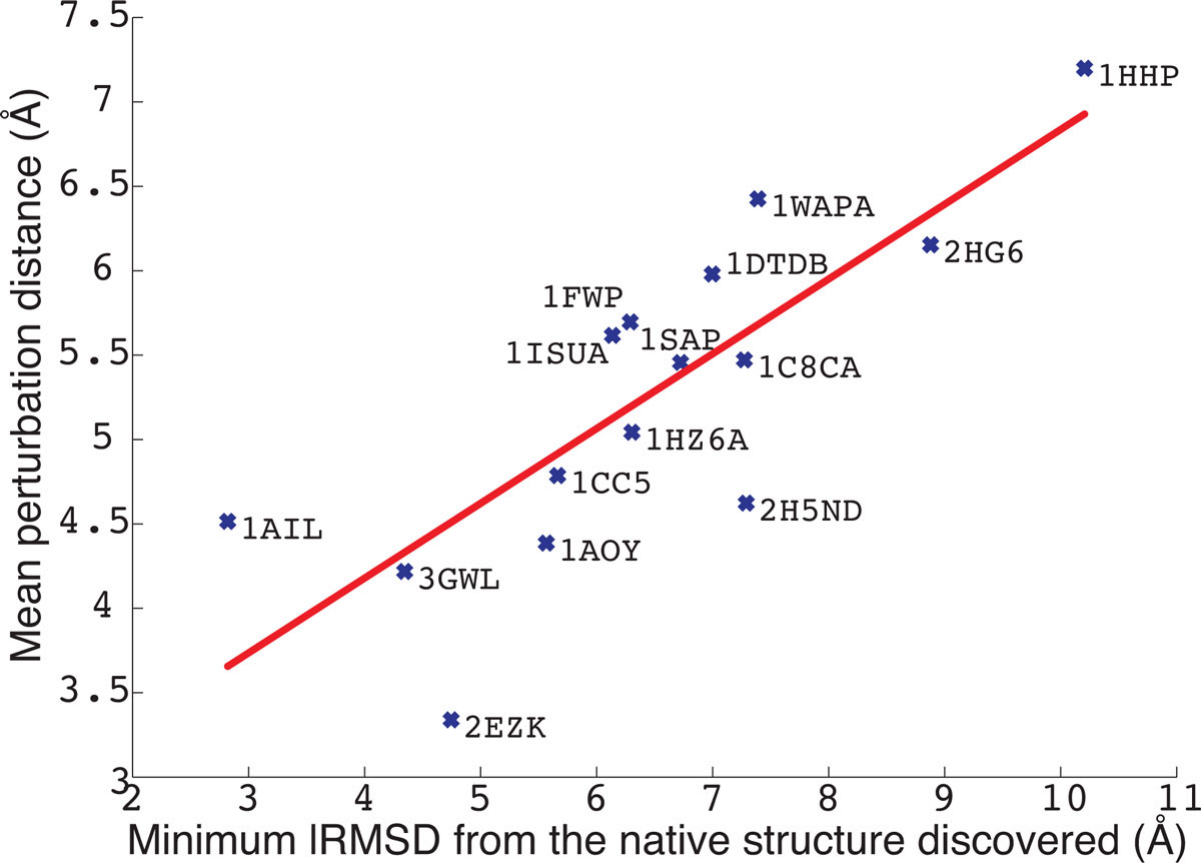
**The mean perturbation distance between *C_i _*and *C_i_*_(perturb)_is plotted against the lowest lRMSD from the native structure obtained by PLOW on each of the 15 protein systems**. The strong linear correlation (the identity line is drawn in red) suggests that the efficacy of the perturbation function is directly related to the efficacy of the search in PLOW.

A very interesting correlation is shown in Figure 4 between the mean lRMSD between *C_i _*and *C_i_*_(perturb) _and the lowest lRMSD between conformations sampled by PLOW and the known native structure in each of the 15 protein systems studied here. The correlation between these two quantities in Figure 4 is about 80%. A lower lRMSD from the native structure corresponds to a smaller jump on average (in terms of lRMSD) made by the perturbation move. This result suggests that the protein systems where PLOW is able to find low lRMSDs to the native structure are also the systems where the perturbation move is able not only to jump out of a current minimum, but also not to jump to a far away region in conformational space. A similar result and observation is attained when correlating the lowest lRMSD to the native structure obtained by PLOW to the mean lRMSD between consecutive local minima in PLOW (lRMSD between *C_i _*and *C_i_*_+1_). Table 3 column 7 shows the mean lRMSD between consecutive local minima in PLOW. The correlation between this distance and the lowest lRMSD to the native structure on the 15 protein systems studied in this paper is even stronger, 90% (data not shown here). Taken together, these results suggest that PLOW performs best when it is able to make no larger than 6Å jumps in terms of mean lRMSD between nearby local minima in the energy surface.

**Figure 5:**
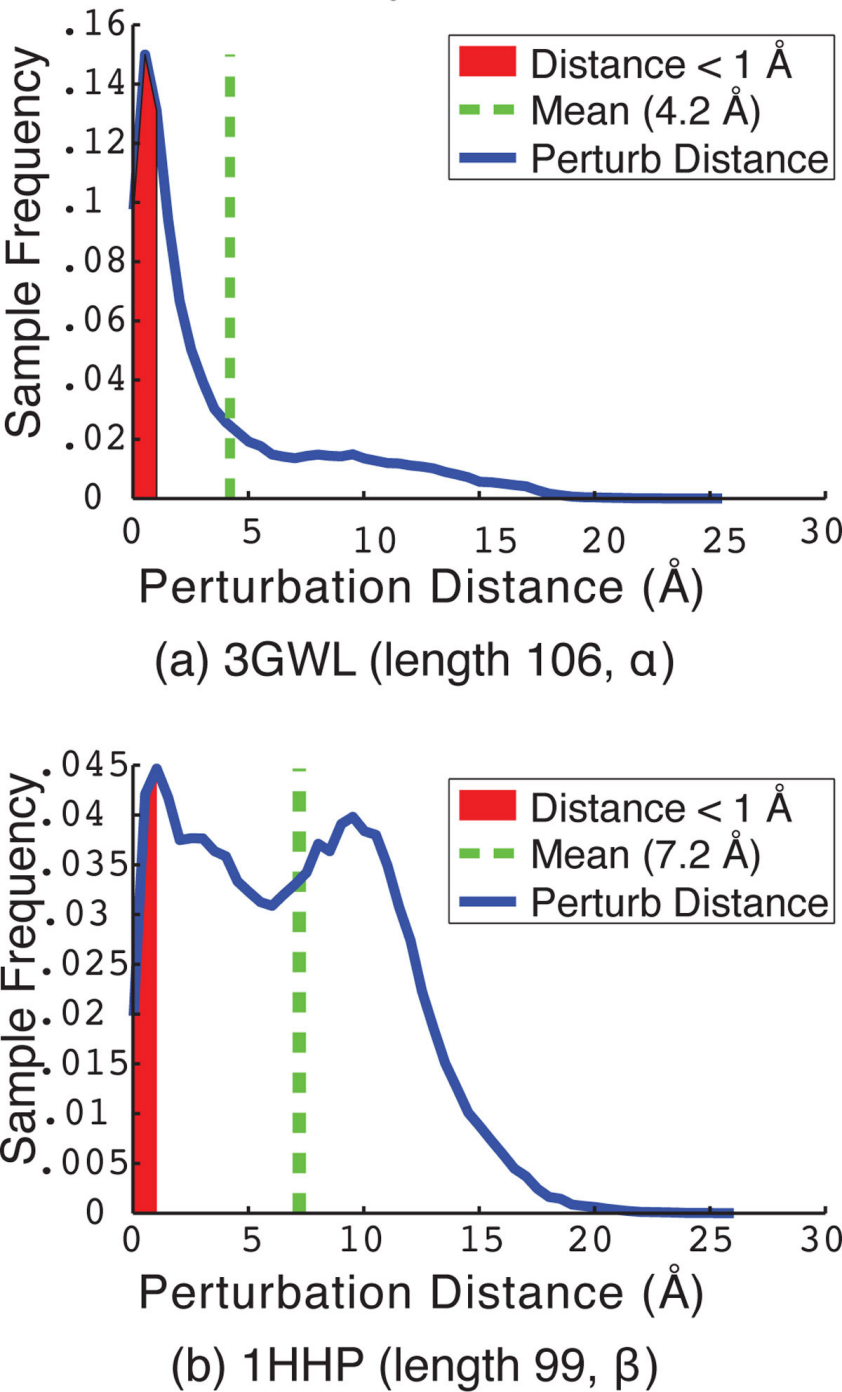
**The distribution of perturbation distances, between *C_i _*and *C_i_*_(perturb)_, is shown for two selected proteins with PDB ids **3GWL **in (a) and 1HHP in (b)**. The area shaded in red represents the cases where the perturbation distance between *C_i _*and *C_i_*_(perturb) _is less than 1Å lRMSD and is thus deemed an insignificant change from the conformation *C_i_*.

A more detailed picture into what the perturbation move is doing is provided in Figure 5, which shows the detailed distribution of lRMSDs between *C_i _*and *C_i_*_(perturb)_for two selected protein systems. The area of the curve shaded in red represents the portion of perturbation moves where this lRMSD is less than 1Å (the move is deemed not to have escaped the current local minimum). The system in Figure 5(a) with native structure PDB id 3GWL is an example of a protein system where PLOW is very effective at finding conformations near the native structure. In this case, the distribution contains a large area of short-to-medium moves with lRMSDs in the 1-8Å range. In contrast, the system in Figure 5(b) with native structure PDB id 1HHP is an example of a system where PLOW does not find conformations near the native structure. Correspondingly, the distribution in Figure 5(b) is weighted towards much higher lRMSDs, with much of the area under the curve above 8Å. This suggests that, in this case, the perturbation move is approaching a random restart.

## Conclusions

The PLOW framework presented in this paper effectively accesses conformations relevant for biological activity in a protein system. PLOW essentially obtains a discrete representation of the relevant conformational space through a set of conformations that map to low-energy local minima in the underlying protein energy surface. PLOW performs a trajectory-based search of the conformational space, where each move in the space is optimized to a corresponding nearby local minimum. Unlike many conformational search algorithms, PLOW explicitly samples local low-energy minima. By focusing on local minima, PLOW results in several fold fewer conformations that can be more manageably analyzed or refined further at greater (atomistic) detail by biophysics studies interested in specific protein systems. While PLOW is useful on a broad range of applications, part of our analysis of its performance employed comparisons of PLOW with conformational search algorithms focused on reproducing the protein native structure. The comparisons show that, by navigating only the space of local minima, PLOW is able to sample conformations near a protein's native structure, either more effectively or as well as state-of-the-art methods. PLOW outperforms our previous FeLTr framework [20] on a diverse set of target proteins. PLOW also performs favorably when compared to published results from two other research groups [18,47]. Comparison of PLOW with an approach that naively samples local minima shows that PLOW accesses a broader set of local low-energy minima, especially on the longer protein chains. Additional analysis of the inner workings of PLOW provides a theoretical basis for the effectiveness of the local minima sampling approach in PLOW and suggests aspects of PLOW that can benefit from further research. Specifically, findings suggest that the perturbation function employed by PLOW is key to its ability to sample a diverse set of low-energy conformations which are more likely to be in proximity of the native structure.

The PLOW framework presented here is a first step towards effective probabilistic search of the protein conformational space. Different implementations can be sought for the algorithmic components identified in PLOW, such as the local search and the perturbation function, in order to obtain different algorithmic realizations of the framework. For instance, the local search can explore uses of MMC and/or employ higher levels of detail through fine-grained representations. Alternative implementations for the perturbation function can incorporate an adaptive temperature schedule. In addition, due to the ability of PLOW to obtain a broad view of the local low-energy minima accessible by a protein chain, novel applications of PLOW will be considered in future work. These applications will not focus on reproducing one native structure but will instead investigate proteins with multiple structurally-diverse functional states.

PLOW is a novel probabilistic search framework that draws inspiration from established search strategies in the evolutionary computation and computational biology communities. The efficacy of PLOW illustrates the benefit in re-examining established methods from other fields which also deal with complex high-dimensional search spaces. The protein conformational space presents unique challenges which go beyond a standard stochastic optimization problem. Combining theoretical findings from the evolutionary computation community with domain-specific knowledge on protein biophysics can result in new powerful approaches. This cross-disciplinary research promises to result in novel powerful search frameworks both for the protein conformational space and generalized optimization problems.

## Competing interests

The authors declare that they have no competing interests.

## Authors' contributions

BSO suggested the methods and the performance study in this manuscript and drafted the manuscript. AS guided the study, provided comments and suggestions on the methods and performance evaluation, and improved the manuscript writing.
